# New Dental Society

**Published:** 1865

**Authors:** 


### NEW DENTAL SOCIETY.

We have just received intimation of a new dental society, organized within a few days, at Louisville, Ky., called "THE CENTRAL STATES DENTAL ASSOCIATION."

It is composed we believe of members of the profession from Kentucky, Tennessee, Indiana and Illinois.

The officers are: President, Dr. W. H. Morgan, Nashville, Tenn.; 1st Vice-President, Dr. S. Driggs, Lexington, Ky.; 2nd Vice-President, Dr. W. G. Redman, Louisville, Ky.; 3d Vice-President, Dr. J. W. Baxter, Warsaw, Ky.; Secretary, Dr. W. H. Shadoan, Louisville, Ky.; Corresponding Secretary, Dr. J. A. McClelland, Louisville, Ky.; Treasurer, Dr. R. H. Wilson Louisville, Ky.

We understand it was a meeting of great interest, and is so organized as to promise permanency and great usefullness. The formation of new societies is a matter of the greatest gratification, and indicates a bright future for the profession.

We designed to have been present at the meeting, and would but for the state of universal sorrow that prevailed at the time; so that we were almost in doubt as to whether there would be a meeting or not. The next meeting will be held in Louisville on THE THIRD TUESDAY OF JULY NEXT.

